# Potential impact of multiple interventions on HIV incidence in a hyperendemic region in Western Kenya: a modelling study

**DOI:** 10.1186/s12879-016-1520-4

**Published:** 2016-04-29

**Authors:** Stéphanie Blaizot, David Maman, Benjamin Riche, Irene Mukui, Beatrice Kirubi, René Ecochard, Jean-François Etard

**Affiliations:** Service de Biostatistique, Hospices Civils de Lyon, F-69003 Lyon, France; Université de Lyon, F-69000 Lyon, France; Université Lyon 1, F-69100 Villeurbanne, France; CNRS UMR 5558, Equipe Biostatistique-Santé, Laboratoire de Biométrie et Biologie Evolutive, F-69100 Villeurbanne, France; Epicentre, F-75011 Paris, France; National AIDS and STDs Control Program, Nairobi, Kenya; Médecins Sans Frontières, Nairobi, Kenya; UMI 233 TransVIHMI, Institut de Recherche pour le Développement, Université Montpellier 1, F-34000 Montpellier, France

**Keywords:** HIV, Hyperendemic settings, Mathematical models, Antiretroviral therapy, Male circumcision, Pre-exposure prophylaxis

## Abstract

**Background:**

Multiple prevention interventions, including early antiretroviral therapy initiation, may reduce HIV incidence in hyperendemic settings. Our aim was to predict the short-term impact of various single and combined interventions on HIV spreading in the adult population of Ndhiwa subcounty (Nyanza Province, Kenya).

**Methods:**

A mathematical model was used with data on adults (15–59 years) from the Ndhiwa HIV Impact in Population Survey to compare the impacts on HIV prevalence, HIV incidence rate, and population viral load suppression of various interventions. These interventions included: improving the cascade of care (use of three guidelines), increasing voluntary medical male circumcision (VMMC), and implementing pre-exposure prophylaxis (PrEP) use among HIV-uninfected women.

**Results:**

After four years, improving separately the cascade of care under the WHO 2013 guidelines and under the treat-all strategy would reduce the overall HIV incidence rate by 46 and 58 %, respectively, vs. the baseline rate, and by 35 and 49 %, respectively, vs. the implementation of the current Kenyan guidelines. With conservative and optimistic scenarios, VMMC and PrEP would reduce the HIV incidence rate by 15–25 % and 22–28 % vs. the baseline, respectively. Combining the WHO 2013 guidelines with VMMC would reduce the HIV incidence rate by 35–56 % and combining the treat-all strategy with VMMC would reduce it by 49–65 %. Combining the WHO 2013 guidelines, VMMC, and PrEP would reduce the HIV incidence rate by 46–67 %.

**Conclusions:**

The impacts of the WHO 2013 guidelines and the treat-all strategy were relatively close; their implementation is desirable to reduce HIV spread. Combining several strategies is promising in adult populations of hyperendemic areas but requires regular, reliable, and costly monitoring.

**Electronic supplementary material:**

The online version of this article (doi:10.1186/s12879-016-1520-4) contains supplementary material, which is available to authorized users.

## Background

Globally, nearly 33 million people are infected with HIV [1]. Although the incidence rate of HIV seems to decrease, the epidemic continues to spread and the situation remains extremely concerning in Sub-Saharan Africa (70 % of the global burden) [1].

Antiretroviral therapy (ART) has greatly reduced HIV-AIDS-related mortality and morbidity in resource-limited countries [2–4] but inequalities in access to therapy still exist [1]. ART is effective in preventing mother-to-child transmission (PMTCT) [5, 6] and sexual transmission of HIV within serodiscordant couples [7–9]. This suggests that increasing ART coverage, thus the number of seropositive patients with undetectable viral loads (VL), should reduce HIV incidence.

Between 2005 and 2007, three clinical trials in South Africa, Kenya, and Uganda have shown that male circumcision may reduce the risk of acquiring HIV by 60 % [10–12]. More recently, community cross-sectional surveys have shown that increasing the prevalence of circumcision among 15–49 years old men was associated with a reduction of HIV incidence as estimated by BED-assays [13]. Besides, since the 2000s, another prevention strategy, pre-exposure prophylaxis (PrEP), is being developed. PrEP is the use of antiretroviral drugs (oral or topical) to prevent HIV acquisition by vulnerable individuals. However, to date, PrEP trials in Sub-Saharan Africa have shown discordant results that depended on the adherence to the treatment [14–19].

Currently, in collaboration with “Médecins Sans Frontières” (Doctors Without Borders), Epicentre is planning a strategy to reduce HIV incidence in Ndhiwa. Ndhiwa is a subcounty of Homa-Bay County in Nyanza region, the Kenyan area the most affected by HIV [20]. In 2012, Epicentre conducted a subcounty-representative cross-sectional population survey, the Ndhiwa HIV Impact in Population Survey (NHIPS), to collect information on the HIV burden in this subcounty; the adult (15–59 years) HIV prevalence was estimated to be 24.1 % [21]. A deterministic compartment-based model calibrated to the NHIPS detailed local data was then built to support operational decisions [22].

In the present study, the model is extended to account for population VL (PVL; i.e., the VL in diagnosed and undiagnosed individuals on ART or not). The aim was to predict the short-term impacts of several (single and combined) interventions on HIV prevalence, HIV incidence, and PVL suppression (i.e., the proportion of HIV-positive individuals with undetectable VL within the whole HIV-positive population) in the adult population (15–59 years old).

## Methods

### Study setting and NHIPS design

Ndhiwa has a population of 172,000 inhabitants of whom nearly 76,880 are aged 15 to 59 years: 42,250 women and 34,630 men. Ndhiwa belongs to Nyanza region which has the highest HIV prevalence rate in Kenya.

The NHIPS is a subcounty-representative cross-sectional population survey conducted from September to November 2012. It used the Demographic and Health Surveys (DHS) [23] methodology to collect information regarding the HIV epidemic in Ndhiwa [21]. The NHIPS consisted of a household questionnaire, an individual questionnaire, and laboratory tests (HIV test, CD4 cell count, assays for recent infection, and VL). Overall, 165 clusters of 20 households were randomly selected. The 3300 successfully interviewed households in the survey included 16,198 persons (8493 women and 7705 men), of whom 6833 were eligible and 6076 agreed to participate. The primary objective of the NHIPS was to estimate HIV incidence using assays for recent infection. The secondary objectives included determining HIV prevalence, the proportion of HIV-positive respondents in need of ART, ART coverage, the proportion of HIV-positive respondents with undetectable VL, HIV testing coverage, the proportion of medically circumcised men, and the access to PMTCT services.

The key HIV indicators (prevalence, incidence, steps of the cascade of care) estimated from the NHIPS have been given elsewhere [21]. Briefly, the overall HIV prevalence was 24.1 % (95 % confidence interval [CI]: 23.0–25.2). This prevalence was higher in women (26.7 %, 95 % CI: 25.3–28.3) than in men (19.8 %, 95 % CI: 18.2–21.6) and was more than two-fold higher in people aged 30–44 years than in those aged 15–29 years (34.7 %, 95 % CI: 32.4–30.8, vs. 16.8 %, 15.4–18.1).

### Model and assumptions

To reach the objectives, we used a modified compartmental model designed to describe HIV transmission, the untreated disease progression, and ART use in the general population [22]. The model split the population into compartments according to sex, age (45 one-year strata from 15 to 59 years), and HIV status: HIV-negative (or susceptible) individuals, untreated HIV-positive individuals with CD4 cell count >350 cells/mm^3^ (compartment I_1_), untreated HIV-positive individuals with CD4 cell count ≤350 cells/mm^3^ (compartment I_2_), and HIV-positive individuals on ART (compartment T). The estimates of the model parameters by sex and age group (15–24, 25–34, 35–59 years) and the estimates of the distributions of the subcounty population among the compartments by sex, age, and HIV status were based on the NHIPS data as described elsewhere [22] (see Additional file 1).

Here, we add information on the PVL to the force of infection: the number of individuals in each compartment was weighted by the proportion of individuals with VL <1000 copies/mL (see Additional file 1). This proportion was considered constant over time: 10 and 8 % in compartment I_1_, 8 and 5 % in compartment I_2_, and 82 and 84 % in compartment T, for women and men, respectively (proportions stemming from the NHIPS). The infectiousness of individuals with VL below this threshold was assumed to be reduced by 96 % [24].

### Interventions modelled

The interventions modelled here are:

#### No change in the current interventions

Here, all the model parameters as estimated by the NHIPS (particularly, treatment and circumcision rates) were considered stable over the four-year simulation time.

#### Improving the cascade of care

This intervention explored the impact of improving the cascade of care with a focus on increasing ART coverage (through expanding the screening and enhancing the linkage to care) and increasing PVL suppression. For this intervention, three ART initiation recommendations were compared: a) the current Kenyan guidelines: CD4 ≤ 350 cells/mm^3^ (PMTCT option A); b) the WHO 2013 guidelines: CD4 ≤ 500 cells/mm^3^ plus PMTCT option B+ (i.e., lifelong ART for all HIV-positive pregnant and breastfeeding women whatever their CD4 cell count) [25]; and, c) treat all HIV-positive subjects whatever the CD4 cell count. In these three recommendations, it is assumed that the overall ART coverage among eligible individuals at the end of the four-year simulation time would reach 80 % (conservative simulation protocol) or 90 % (optimistic simulation protocol; Table 1). To do this in the three recommendations, the transition rate (or flow) between compartments I_2_ and T was changed. Moreover, in settings b and c, a transition rate was added between compartments I_1_ and T and its value was assumed to increase linearly over the first year and finally equal the transition rate between compartments I_2_ and T; in setting b, this transition rate was multiplied by the proportion of individuals with CD4 cell counts ≤500 cells/mm^3^ or pregnant or breastfeeding HIV-positive women (proportions stemming from the NHIPS).

**Table 1:** Baseline coverage of the interventions stemming from the NHIPS (2012) and simulation protocols of the interventions modelled

Interventions modelled	Baseline	Simulation protocol	
		Conservative	Optimistic
Antiretroviral therapy coverage among eligible subjects (%)			
CD4 ≤ 350 cells/mm^3^	69 %	80 %	90 %
CD4 ≤ 500 cells/mm^3^ or PMTCT option B+	51 %	80 %	90 %
All-CD4	40 %	80 %	90 %
Voluntary medical male circumcision among uninfected men			
15–24 years	42 %	75 %	90 %
25–34 years	17 %	30 %	70 %
35–59 years	5 %	10 %	60 %
All	25 %	50 %	80 %
Pre-exposure prophylaxis among uninfected women			
15–24 years	-	60 %	80 %
25–34 years	-	10 %	30 %
35–59 years	-	10 %	20 %
All	-	34 %	51 %

*PMTCT B+* Prevention of mother-to-child transmission of HIV with lifelong ART for all-HIV-positive pregnant and breastfeeding women whatever their CD4 cell count

#### Voluntary medical male circumcision (VMMC)

This intervention assumed that, overall, 50 % or 80 % (conservative or optimistic simulation protocol, respectively) of HIV-negative men would be circumcised at the end of the four-year simulation time with a linear increase over time; however, these proportions were given various values depending on the age group (Table 1). The protective effect of circumcision was assumed to reduce female–male transmission by 60 % [10–12].

#### Pre-exposure prophylaxis (PrEP) in women

This intervention assumed that, overall, 34 % or 51 % (conservative or optimistic simulation protocol, respectively) of HIV-negative women would use PrEP at the end of the four-year simulation time with a linear increase over time; however, these proportions were given various values depending on the age group (Table 1). The protective effect of PrEP was assumed to reduce male–female transmission by 50 %.

#### Combined interventions

In the first combined intervention, the WHO 2013 guidelines were combined with VMMC (“ART & VMMC”). In the second, the WHO 2013 guidelines were combined with both VMMC and PrEP (“ART, VMMC, & PrEP”). In these combinations, the conservative (or optimistic) simulation protocol kept the conservative (or optimistic) simulation protocol of each single intervention. For comparison purposes, we combined also the current Kenyan guidelines with VMMC as well as the treat-all strategy with VMMC.

These interventions were compared with respect to different predicted outcomes: the prevalence, the incidence rate, the incidence rate ratio (i.e., the incidence rate at end of the four-year simulation time divided by the incidence rate at baseline), and the PVL suppression reached after four years. PVL suppression was defined as the proportion of HIV-positive subjects with a VL <1000 copies/mL (with the use of the compartment-specific proportions given in “Model and assumptions”).

## Results

### Single interventions

**Fig. 1:**
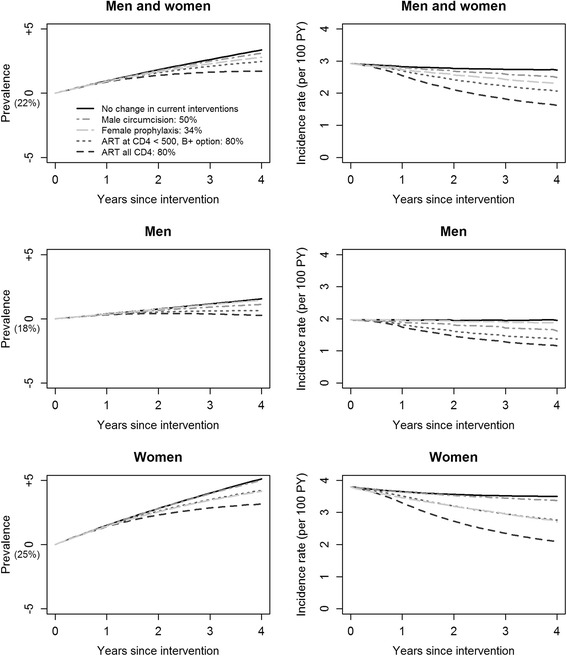
Short-term changes in HIV prevalence and incidence rates of single interventions with conservative simulation protocols

**Fig. 2:**
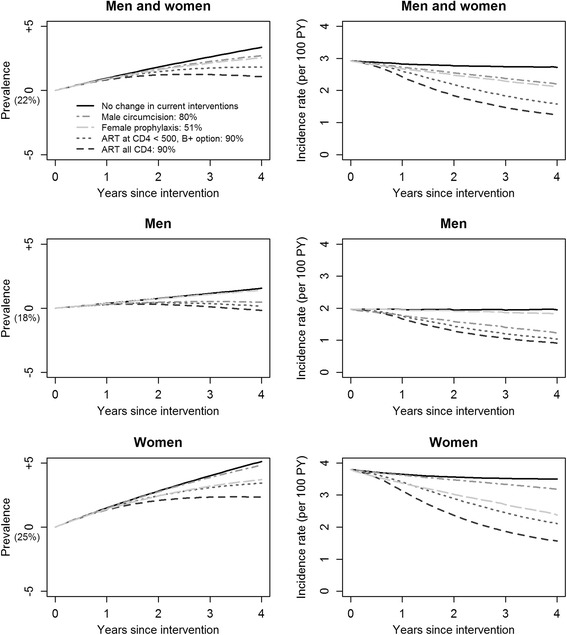
Short-term changes in HIV prevalence and incidence rates of single interventions with optimistic simulation protocols

In the short term, under scenario “No change in the current interventions”, the HIV incidence rate would be reduced by 7 % (1 % in men and 8 % in women) whereas the prevalence would reach 25.3 % (19.1 % in men and 30.6 % in women). In the short term, with both conservative and optimistic simulation protocols, the treat-all strategy would have the greatest impact on reducing HIV incidence rate in men (by 41 and 54 %, respectively) and women (by 45 and 59 %, respectively; Figs. 1 and 2) followed by the WHO 2013 guidelines in men (by 31 and 47 %) and women (by 27 and 44 %). VMMC would also have a great impact on reducing the incidence rate (by 15 and 25 %) but a higher impact among men (the direct target) than among women (18 and 38 % vs. 11 and 16 % reduction, respectively). PrEP would also have a great impact on the overall HIV incidence (by 22 and 28 %); however, it would have a higher impact among women (the direct target) than among men (29 and 37 % vs. 6 and 8 % reduction). All interventions would slightly increase HIV prevalence over the four years (1.0–1.2x the baseline) but this increase would be the lowest with the treat-all strategy.

Considering a 90 % ART coverage, the treat-all strategy would have a higher impact on the HIV incidence rate than the WHO 2013 guidelines or the current Kenyan guidelines, the reduction being respectively 58, 46, and 17 % vs. the baseline rate (see Additional file 1: Figure A2).

In the NHIPS, the baseline PVL suppression was about 37 %. The treat-all strategy over four years would cause the greatest PVL suppression: 68 and 75 % respectively with the conservative and optimistic simulation protocols vs. 47 % with no change in the current interventions (see Additional file 1: Figure A3). The WHO 2013 guidelines would lead to 60 and 70 % PVL suppression, respectively.

**Fig. 3:**
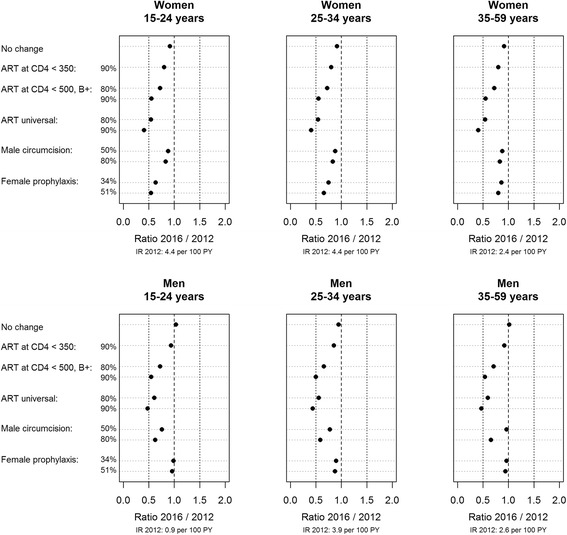
Predicted incidence rate ratios after four years of single interventions by sex and age group

Depending on the sex and the age group, the treat-all strategy would cause the greatest reduction in the HIV incidence rate (39 to 46 % with the conservative simulation protocol and 52 to 59 % with the optimistic simulation protocol), followed by the WHO 2013 guidelines (28 to 34 % with the conservative simulation protocol and 45 to 50 % with the optimistic simulation protocol, Fig. 3). VMMC would reduce the HIV incidence rate in men: 4 to 24 % in the conservative simulation protocol and 35 to 41 % in the optimistic simulation protocol. PrEP would reduce HIV incidence rate in women: 14 to 37 % with the conservative simulation protocol and 20 to 46 % with the optimistic simulation protocol, mostly in the targeted 15–24 age group. The current Kenyan guidelines would have a modest short-term impact on the HIV incidence rate (a 6 to 20 % reduction vs. the baseline) because the baseline ART coverage is already close to 70 %.

### Combined interventions

Combined interventions would have greater impacts on HIV prevalence and incidence rate than single interventions. Precisely, “ART, VMMC, & PrEP” would have the greatest impact on the overall incidence rate (46 and 67 % reduction in the conservative and optimistic simulation protocol, respectively, vs. the baseline rate; see Additional file 1: Figure A4). “ART & VMMC” would have a great impact on the incidence rate (35 and 56 % reduction in the conservative and optimistic simulation protocol, respectively, vs. the baseline rate).

**Fig. 4:**
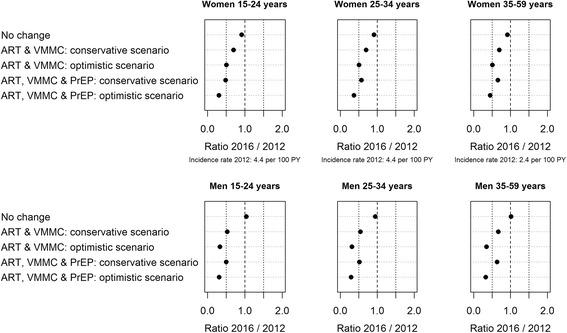
Predicted incidence rate ratios after four years of combined interventions by sex and age group. “ART & VMMC” included “cascade of care” intervention under the WHO 2013 guidelines combined with voluntary medical male circumcision (VMMC). “ART, VMMC & PrEP” included “cascade of care” intervention under the WHO 2013 guidelines with VMMC and female pre-exposure prophylaxis

Depending on the sex and the age group, “ART, VMMC, & PrEP” would have the greatest impact on the HIV incidence rate; it would reduce it by 34 to 52 % with the conservative simulation protocol and by 55 to 71 % with the optimistic simulation protocol vs. the baseline rate (Fig. 4). “ART & VMMC” would reduce the incidence rate by 30 to 47 % with the conservative simulation protocol and by 49 to 69 % with the optimistic simulation protocol vs. the baseline rate.

Over four years, the incidence rate would be reduced by 24 and 33 % with the combination of the current Kenyan guidelines plus VMMC and by 49 and 65 % with the combination of a treat-all strategy plus VMMC.

## Discussion

A deterministic compartmental model was used to investigate the short-term impacts of various single and combined interventions on HIV incidence in the adult population (15–59 years old) of Ndhiwa subcounty, Kenya.

Our results showed that an improvement of the “cascade of care” according to the WHO 2013 guidelines or a treat-all strategy would have a high impact on the HIV incidence rate (46 and 58 % reduction vs. the baseline rate). The treat-all strategy would have a greater impact among women than among men whereas the WHO 2013 guidelines would have a greater impact among men than among women; this is due to the fact that, in the latter case, a higher proportion of women are eligible for ART, which results in a lower weighted prevalence of HIV in women than in men. In comparison with the current Kenyan guidelines, the optimistic simulation protocol with the WHO 2013 guidelines and the treat-all strategy would result in a 35 % and 49 % incidence reduction, respectively, after four years. Previous mathematical models applied to Sub-Saharan populations have shown that long-term ART interventions (especially ‘test and treat’ strategy) would substantially reduce the HIV incidence rate [26–33].

Here, the impacts of improving the “cascade of care” under the WHO 2013 guidelines and those of the treat-all strategy were relatively close. This may be explained by the high proportion of eligible individuals under the WHO 2013 guidelines in NHIPS (81 % of all HIV-positive individuals) [21]. Moreover, the time from infection to reaching a CD4 cell count <500 cells/mm^3^ has been estimated to be relatively short [34]. This would result in quick eligibility of individuals for ART. In addition, another modelling study showed close impacts of the treat-all strategy and an extension of the eligibility for ART (from <350 to <500 CD4 cells/mm^3^): using several mathematical models, it has been shown that, in South Africa, this extension would prevent 5 to 12 % new infections whereas an extension to all HIV-positive individuals would prevent 9 to 32 % new infections over 20 years depending on the mathematical model and assuming no additional testing and linkage to care [35]. These results suggest that whatever the strategy (treat-all or treat subjects with <500 CD4 cells/mm^3^), the main effort would still be to test and offer treatment.

Our results showed that, in the short term, the PVL suppression would reach 60 to 70 % under the WHO 2013 guidelines or 68 to 75 % under the treat-all strategy, depending on the simulation protocol, whereas it would be 47 % with no change in the current interventions. To our knowledge, no previous modelling study has calculated this metric yet. Actually, this metric is interesting because it has been shown to be negatively associated with incidence [36–38] and can be more easily estimated than incidence. Moreover, this metric will be increasingly used with the potential future availability of point-of-care viral load tests in resource-limited settings.

The treat-all strategy could simplify the strategies aiming at increasing ART coverage but would raise ethical questions (ART for “healthy” people), long-term toxicity, viral resistance issues, and cost issues. However, initiating ART at <500 CD4 cells or even earlier has shown individual clinical benefits [39–41] and recent models have shown that extending ART eligibility to <500 CD4 cells/mm^3^ or to all HIV-positive individuals would be cost-effective [35]. In fact, randomized controlled trials are currently addressing the issues of acceptability and feasibility of offering early ART and the impact of early treatment on HIV incidence [42].

Our results suggest that VMMC alone would reduce the overall incidence rate by 15 to 25 %, depending on the simulation protocol. Its impact is expected to be higher in men than in women because the former are directly protected by circumcision. Previous mathematical models applied to Sub-Saharan populations showed that, in the long-term, circumcision would also benefit indirectly to women and uncircumcised men [43–47].

The present results suggest that female PrEP, as a single intervention, would reduce the overall HIV incidence rate by 22 to 28 % according to the simulation protocol. This intervention was included here despite its costs and previous mixed results in Sub-Saharan Africa (discrepancies explained by differences in adherence levels [19]). Previous models of PrEP applied to Sub-Saharan African countries have shown short-, middle-, and long-term protection against new HIV infections in the general population [48–50] and in high-risk populations (female sex workers and clients [51], people with high sexual activity [48], young people [48], young women [49] or serodiscordant couples [52]). Some models have also shown that PrEP may be cost-effective in hyperendemic settings [49, 50, 52].

The present results show that combining the WHO 2013 guidelines with VMMC would reduce the HIV incidence rate by 35 to 56 % according to the simulation protocol and that combining treat-all with VMMC would reduce HIV incidence by 49 to 65 %. These results are consistent with those of a recent modelling of data from KwaZulu-Natal (South Africa); i.e., a 63 % reduction in the HIV incidence rate within four years with a combination of four interventions: increasing the coverage of testing and counselling, reducing risky behaviour, increasing the coverage of VMMC, and increasing ART coverage (with a ‘test and treat’ strategy) [53]. Recently, Cori et al. estimated that a combination of increasing home-based voluntary testing and counselling, increasing VMMC coverage, and increasing ART coverage with a treat-all strategy over three years would lead to a 60 % reduction vs. no change in the current interventions [54]. Here, making the same calculation, the WHO 2013 guidelines with VMMC would lead to a 37 to 53 % reduction. Combining the WHO 2013 guidelines with VMMC and PrEP would reduce the HIV incidence rate by 46 to 67 %. Previous models have shown that combining ART and PrEP would have a higher impact on HIV incidence than ART alone [52, 55].

The strengths of our study are: i) the calibration of a model on the basis of detailed local and recent population-based data (including individual CD4 measurements); ii) the availability of viral load measurements at the population level (i.e., in both diagnosed and undiagnosed individuals, under treatment or not), making it possible to estimate the PVL suppression in the short term after various interventions; and, iii) the intervention scenarios were based on the current levels of coverage of these interventions by sex and by age classes.

Our analysis had some limitations. First, the various steps in the cascade of care were implicitly modelled; however, the success of an ART intervention depends on each step of the cascade of care and, at each step, cohort attrition may occur because of insufficient screening policy, fear of diagnosis or treatment, lack of access to ART, etc. [56]. Second, in our optimistic simulation protocol, the proportion of HIV-negative men to circumcise in age group 35–59 years is relatively high; however, not all men would accept circumcision. Actually, in the ANRS-12126 Male Circumcision Project, the proportion of circumcised men raised over three years from 6 % in age group 35–39 years and 17 % in age group 40–49 years to 37 and 34 %, respectively [13]; this suggests that men in age group 35–49 years are likely to accept circumcision. Third, risk compensation was not modelled. Risk compensation could reduce the benefit of the interventions at the population level. Some models relative to circumcision, as a single intervention, have shown that a high increase in risky behaviour may reduce the benefits from VMMC regarding HIV prevalence or incidence [44–46, 57, 58]. Fourth, we did not consider drug resistance in HIV-positive individuals on ART or in individuals using PrEP; indeed, drug resistance can also reduce the benefit of ART and PrEP at the population level. Mathematical models have shown that an increase in ART coverage would increase the prevalence of drug resistance in resource-limited settings [27, 28, 59]. Recent models including ART and PrEP suggest that the majority of resistance cases were rather due to ART than to PrEP, ART coverage being higher than PrEP coverage [55, 60].

The likelihood of success of a HIV prevention or treatment intervention may be increased by door-to-door home-based interventions with voluntary HIV testing and counselling and with the support of the community. Depending on the HIV status, counselling may target VMMC or/and focus on HIV care and referral to care clinics. This type of campaign has been associated with high ART coverage and high VMMC prevalence in a rural/semi-urban area in Uganda [61] and with high ART coverage and high level of PVL suppression in rural KwaZulu-Natal [62]. Such a campaign may be successfully implemented in Ndhiwa given the high level of participation in the NHIPS survey (response rate close to 90 %) [21].

## Conclusions

The implementation of the WHO 2013 guidelines is expected to be efficient in reducing the spread of HIV. Combining strategies is promising in reducing HIV incidence in adult populations of hyperendemic areas, but requires careful monitoring over time.

### Ethics approval and consent to participate

Ethical approval was obtained in Kenya from the Kenya Medical Research Institute Ethical Review Committee (KEMRI, ref 347) and in France from the “Comité de Protection des Personnes d’Ile de France” (CPP, ref 12056). Written consent for participating in the study and undergoing HIV testing was obtained from each participant prior to the survey interview.

### Availability of data and materials

The readers interested in using the NHIPS data may contact D. Maman at Epicentre, 8 rue Saint Sabin, F-75010 Paris, France.

## Additional file

Additional file 1: Appendix. Technical appendix and supplementary figures. (PDF 685 kb)

## Abbreviations

AIDS: acquired immunodeficiency syndrome
ART: antiretroviral therapy
DHS: Demographic and Health Surveys
HIV: human immunodeficiency virus
NHIPS: the Ndhiwa HIV Impact in Population Survey
PMTCT: prevention of mother-to-child transmission
PrEP: pre-exposure prophylaxis
PVL: population viral load
VL: viral load
VMMC: voluntary medical male circumcision
WHO: World Health Organization
