# Supplementary material for: Potential impact of multiple interventions on HIV incidence in a hyperendemic region in Western Kenya: a modelling study
Source: BMC Infect Dis. 2016 Apr 29;16:189. doi: 10.1186/s12879-016-1520-4 (PMC4851795; doi:10.1186/s12879-016-1520-4)
Supplement: Additional file 1: — Appendix. Technical appendix and supplementary figures. (PDF 685 kb) [file 12879_2016_1520_MOESM1_ESM.pdf]

Appendix

Potential impact of multiple interventions on HIV incidence in a hyperendemic region in Western Kenya: a modelling study

Stéphanie BLAIZOT, David MAMAN, Benjamin RICHE, Irene MUKUI, Beatrice KIRUBI, René ECOCHARD, Jean-François ETARD

Mathematical model

A detailed description of the mathematical model and the estimations of the model parameters can be found in Reference 1 [1]. Here, we described briefly the main features of the mathematical model and show the way PVL was taken into account in the force of infection.

The model, shown in Figure A1, describes HIV transmission, the untreated-disease progression, and ART use in a heterosexual population. It splits the population into compartments according to sex, age, and HIV status. The population of infected individuals was split into three compartments according to the CD4 cell count and the ART status: 1) Compartment I_1: untreated HIV-positive individuals with CD4 cell count >350 cells/mm^3; 2) Compartment I_2: untreated HIV-positive individuals with CD4 cell count ≤350 cells/mm^3 (immunosuppressed individuals); and, 3) Compartment T: HIV-positive individuals under ART. An additional Compartment S was dedicated to HIV-negative (or susceptible) individuals.

Notation

S: susceptible individuals of the studied population

I_1: HIV-positive and untreated individuals with CD4 cell count >350 cells/mm3

I_2: HIV-positive and untreated individuals with CD4 cell count ≤350 cells/mm3

T: HIV-positive individuals on ART

D: deceased individuals

λ_S: force of infection

λ_I: immunosuppression rate

λ_T: treatment rate

μ_S: mortality rate of individuals in compartment S

μ_{I1}: mortality rate of individuals in compartment I_1

μ_{I2}: mortality rate of individuals in compartment I_2

μ_T: mortality rate of individuals in compartment T

Differential equations

We formulated the predictive model as a system of sex and age-specific (a = 15, ...59 years) differential equations:

$$\begin{cases} \dfrac{dS_{Sex,a}}{dt} = -\lambda_{S,Sex,a}\ S_{Sex,a} - \mu_{S,Sex,a}\ S_{Sex,a} - \nu\ S_{Sex,a} + \nu\ S_{Sex,a-1} \\ \dfrac{dI_{1Sex,a}}{dt} = \lambda_{S,Sex,a}\ S_{Sex,a} - \left(\mu_{I_1,Sex,a} + \lambda_{I,Sex,a}\right) I_{1Sex,a} - \nu\ I_{1Sex,a} + \nu\ I_{1Sex,a-1} \\ \dfrac{dI_{2Sex,a}}{dt} = \lambda_{I,Sex,a}\ I_{1Sex,a} - \left(\mu_{I_2,Sex,a} + \lambda_{T,Sex,a}\right) I_{2Sex,a} - \nu\ I_{2Sex,a} + \nu\ I_{2Sex,a-1} \\ \dfrac{dT_{Sex,a}}{dt} = \lambda_{T,Sex,a}\ I_{2Sex,a} - \mu_{T,Sex,a}\ T_{Sex,a} - \nu\ T_{Sex,a} + \nu\ T_{Sex,a-1} \end{cases}$$

Ageing was considered through the last two terms of each equation; ν being the rate at which an individual moves from one age class to another. For the first age class (15 years), ageing was taken into account as follows: at one year intervals, a fixed number of individuals were put into compartment S.

The force of infection is frequency-dependent; it included HIV prevalence in the opposite sex weighted by the infectiousness of HIV-positive individuals. This infectiousness (probability of transmitting the virus) depends on the viral load. This force of infection may then be written as follows:

$$\lambda_{S,Sex,a} = \tilde{\beta}_{Sex,a} \left(\frac{J_{OppositeSex}}{N_{OppositeSex}} \right) \zeta_{Sex,a}$$

with:

$\tilde{\beta}_{Sex,a}$ the transmission parameter;

$\zeta_{Sex,a} = (1-\varphi_{Sex,a}) + \gamma\,\varphi_{Sex,a}$ allows us to take into account that some individuals, in proportion φ, have a different susceptibility (reduced by γ) due to, for example, circumcision (in men);

$$\begin{aligned}J_{OppositeSex} = I_{1OppositeSex}&\left[\left(1-propVLlow_{I_1,OppositeSex}\right)+propVLlow_{I_1,OppositeSex}\cdot\varepsilon\right]+\\ I_{2OppositeSex}&\left[\left(1-propVLlow_{I_2,OppositeSex}\right)+propVLlow_{I_2,OppositeSex}\cdot\varepsilon\right]+\\ T_{OppositeSex}&\left[\left(1-propVLlow_{T,OppositeSex}\right)+propVLlow_{T,OppositeSex}\cdot\varepsilon\right]\end{aligned}$$

$propVLlow_{X,OppositeSex}$ is the (baseline) proportion of individuals with a viral load below 1000 copies/mL in the compartment *X* and ε is the reduction of infectiousness in individuals with a viral load below 1000 copies/mL.

Calibration

The model parameters are the following: i) the force of infection (λ_S); ii) the "immunosuppression rate" (λ_I); i.e., the rate at which an individual moves from > 350 to ≤ 350 cells/mm^3 CD4 cell count; iii) the "treatment rate" (λ_T) or the ART initiation rate; and, iv) the mortality rate (μ).

Estimating the model parameters included three stages. First, we used the HIV status, the self-reported ART status, and the CD4 cell count at the time of the survey to assign each individual to one of the above-cited compartments. Second, we derived individuals' states (as described by the compartments) during the past year from the individuals' histories (self-reported dates of first positive HIV test, last HIV test and its result, and ART initiation) and CD4 measurement. We used only the previous year to minimize the recall bias and avoid

making too strong assumptions about individuals' histories. Third, we calculated the number of transitions between pairs of compartments and the time spent by each individual in each compartment. The model parameters are given in Table A1.

The initial compartment sizes (i.e., the number of individuals in each compartment) stemming from the NHIPS survey (applied on the 2009 Ndhiwa District census) and the parameters provided by the estimation step were used to predict the short-term course of the HIV epidemic in the adult population (15-59 years old).

References

1. Blaizot S, Riche B, Maman D, Mukui I, Kirubi B, Etard JF, *et al.* Estimation and Short-Term Prediction of the Course of the HIV Epidemic Using Demographic and Health Survey Methodology-Like Data. *PLoS One* 2015; **10(6)**:e0130387.

Additional table

Table A1. Estimated model parameters and their 95% confidence intervals (per 1000 person-years).

Parameter	Women	Men
Infection rate *(λ_S)*		
15-24 years	47 [35 - 63]	9 [4 - 19]
25-34 years	48 [33 - 69]	41 [24 - 67]
35-59 years	26 [17 - 40]	27 [16 - 46]
Immunosuppression rate *(λ_I)*		
15-24 years	153 [75 - 312]	0*
25-34 years	207 [132 - 323]	335 [170 - 660]
35-59 years	198 [116 - 338]	216 [113 - 412]
Treatment rate *(λ_T)*		
15-24 years	439 [236 - 815]	519 [61 - 4440]
25-34 years	480 [337 - 683]	334 [169 - 659]
35-59 years	793 [576 - 1092]	631 [461 - 863]
Mortality rate among subjects with > 350 CD4 cells/mm^3 *(μ_S, μ_{I1}, μ_T)*		
15-19 years	1.45 [0.97 - 1.93]	2.31 [1.68 - 2.93]
20-24 years	2.90 [2.27 - 3.53]	2.92 [2.26 - 3.58]
25-29 years	4.81 [4.00 - 5.62]	3.58 [2.86 - 4.30]
30-34 years	4.69 [3.89 - 5.48]	6.61 [5.50 - 7.72]
35-39 years	6.75 [5.59 - 7.90]	6.69 [5.49 - 7.90]
40-44 years	8.18 [6.61 - 9.74]	11.03 [9.01 - 13.05]
45-59 years	8.70 [6.42 - 10.98]	13.42 [10.55 - 16.29]
Mortality rate among subjects with ≤ 350 CD4 cells/mm^3 *(μ_{I2})*		
15-19 years	9.38 [6.27 - 12.48]	27.39 [19.94 - 34.83]
20-24 years	9.67 [7.58 - 11.76]	13.31 [10.32 - 16.30]
25-29 years	15.16 [12.60 - 17.73]	12.23 [9.77 - 14.69]
30-34 years	25.45 [21.14 - 29.76]	14.53 [12.09 - 16.97]
35-39 years	32.10 [26.60 - 37.60]	22.85 [18.73 - 26.98]
40-44 years	28.74 [23.24 - 34.24]	32.62 [26.63 - 38.61]
45-59 years	32.44 [23.94 - 40.94]	48.56 [38.17 - 58.94]

* Value estimated at 0 because no transition between compartments I_1 and I_2 was observed in this group.

Additional figures

Figure A1. Flow diagram of the model.

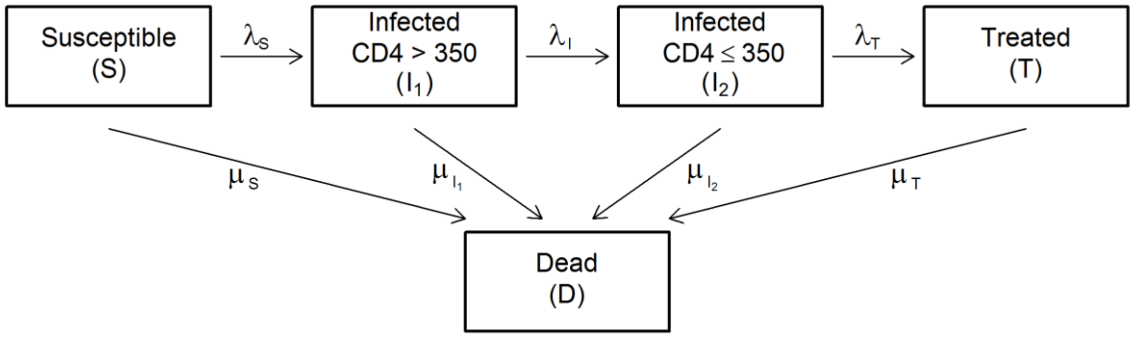

Figure A2. Comparison of predicted incidence rate ratios (2016 over 2012) according to ART initiation guidelines.

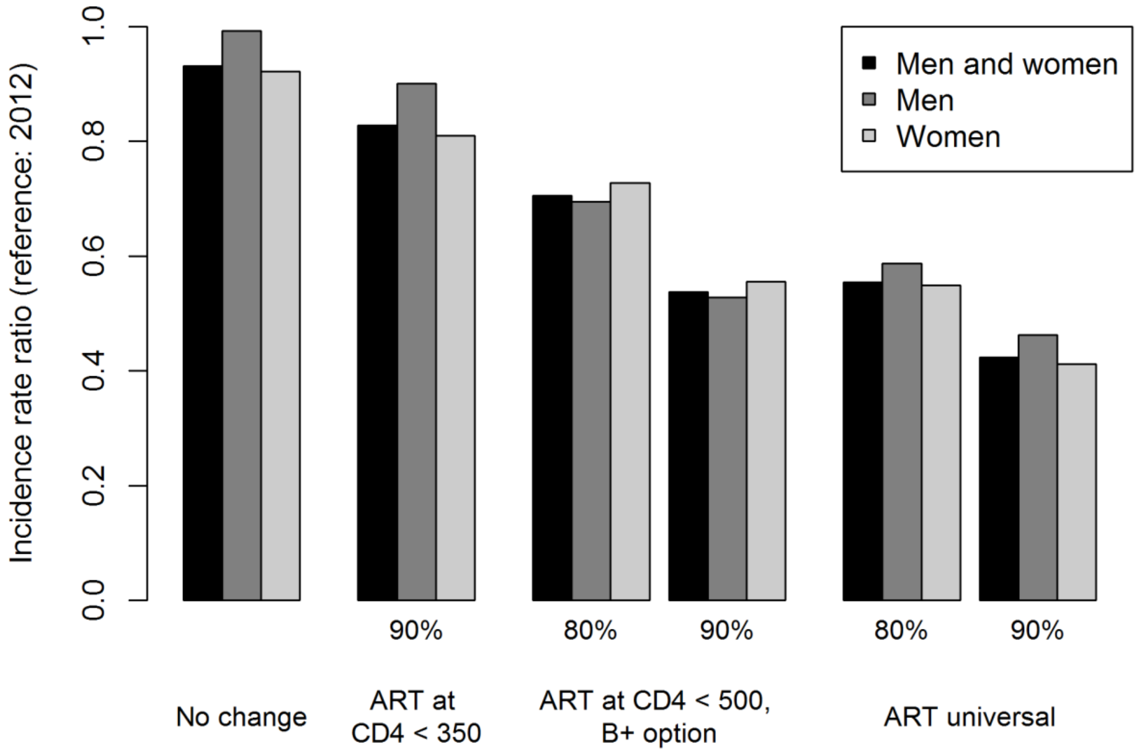

Figure A3. Simulation of population viral load suppression after four years of single interventions.

The black bar is the baseline population viral load calculated in the NHIPS; the grey bars are the population viral load calculated using the simulations after four years of interventions.

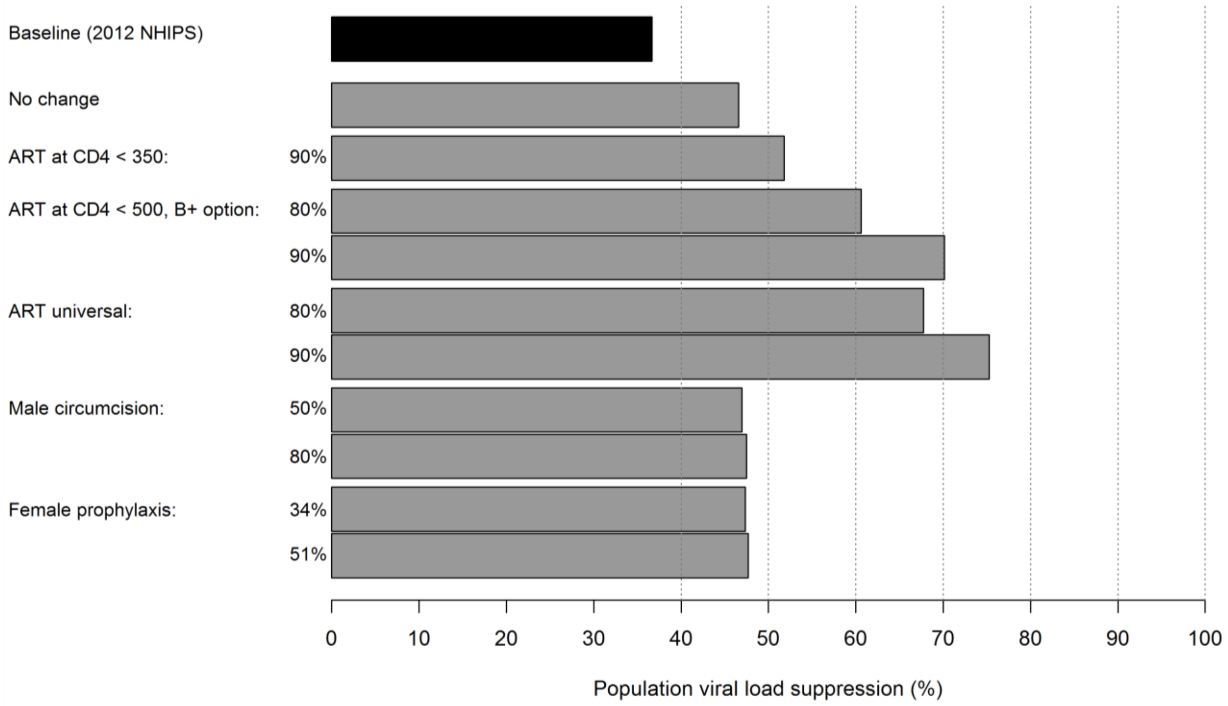

Figure A4. Short-term changes in HIV prevalence and incidence rates of combined interventions.

"ART & VMMC" included "cascade of care" intervention under the WHO 2013 guidelines combined with voluntary medical male circumcision (VMMC). "ART, VMMC & PrEP" included "cascade of care" intervention under the WHO 2013 guidelines with VMMC and female pre-exposure prophylaxis.

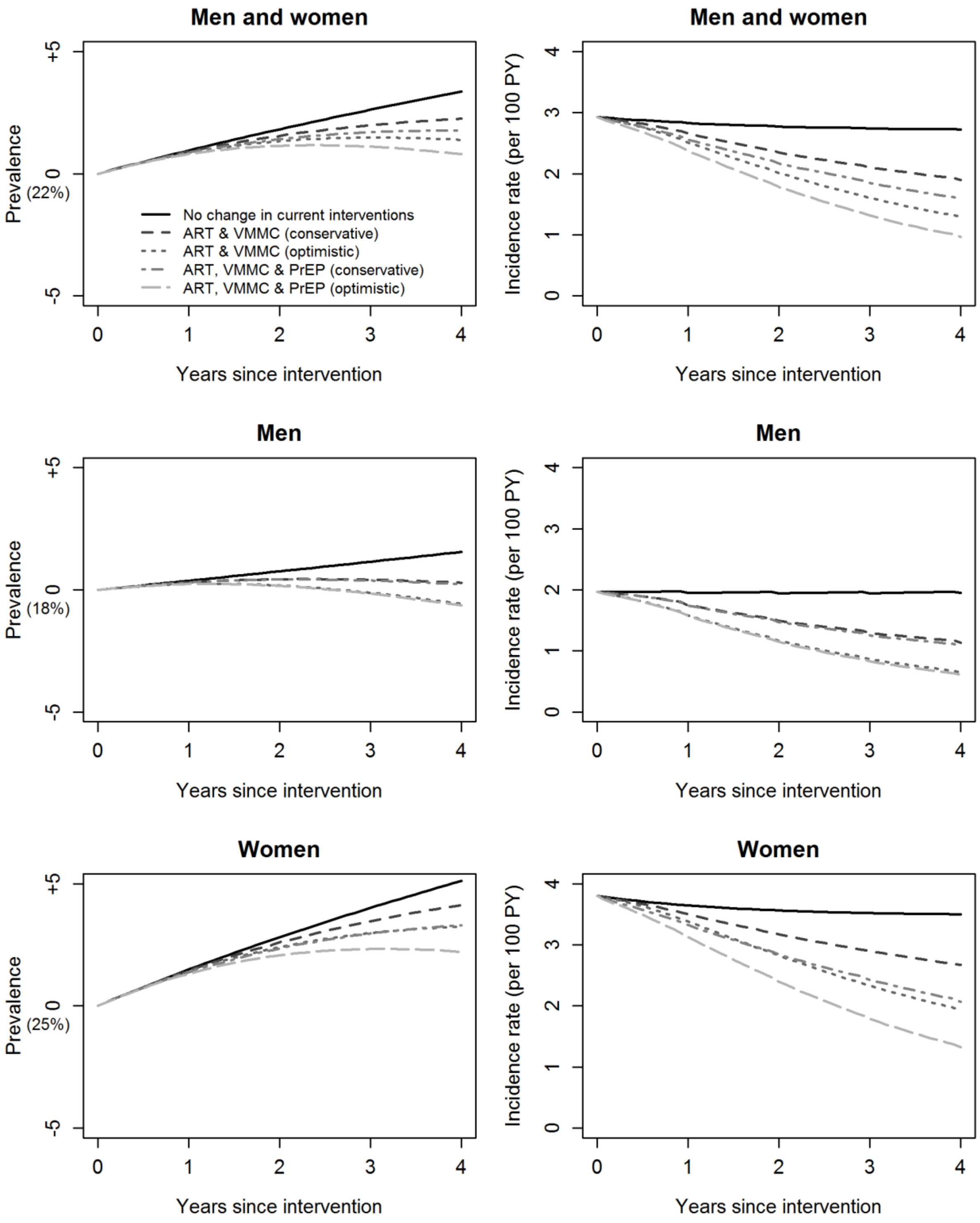
Men and women
Prevalence
(22%)
+5
0
-5
No change in current interventions
ART & VMMC (conservative)
ART & VMMC (optimistic)
ART, VMMC & PrEP (conservative)
ART, VMMC & PrEP (optimistic)
Years since intervention
Incidence rate (per 100 PY)
Men
(18%)
Women
(25%)
0
1
2
3
4
